# Targeting Stress Erythropoiesis Pathways in Cancer

**DOI:** 10.3389/fphys.2022.844042

**Published:** 2022-05-25

**Authors:** Sanja Vignjević Petrinović, Aleksandra Jauković, Maja Milošević, Diana Bugarski, Mirela Budeč

**Affiliations:** ^1^ Laboratory for Neuroendocrinology, Institute for Medical Research, National Institute of Republic of Serbia, University of Belgrade, Belgrade, Serbia; ^2^ Laboratory for Experimental Hematology and Stem Cells, Institute for Medical Research, National Institute of Republic of Serbia, University of Belgrade, Belgrade, Serbia

**Keywords:** stress erythropoiesis, erythroid progenitors, anemia, cancer, erythropoietin

## Abstract

Cancer-related anemia (CRA) is a common multifactorial disorder that adversely affects the quality of life and overall prognosis in patients with cancer. Safety concerns associated with the most common CRA treatment options, including intravenous iron therapy and erythropoietic-stimulating agents, have often resulted in no or suboptimal anemia management for many cancer patients. Chronic anemia creates a vital need to restore normal erythropoietic output and therefore activates the mechanisms of stress erythropoiesis (SE). A growing body of evidence demonstrates that bone morphogenetic protein 4 (BMP4) signaling, along with glucocorticoids, erythropoietin, stem cell factor, growth differentiation factor 15 (GDF15) and hypoxia-inducible factors, plays a pivotal role in SE. Nevertheless, a chronic state of SE may lead to ineffective erythropoiesis, characterized by the expansion of erythroid progenitor pool, that largely fails to differentiate and give rise to mature red blood cells, further aggravating CRA. In this review, we summarize the current state of knowledge on the emerging roles for stress erythroid progenitors and activated SE pathways in tumor progression, highlighting the urgent need to suppress ineffective erythropoiesis in cancer patients and develop an optimal treatment strategy as well as a personalized approach to CRA management.

## Introduction

Anemia commonly occurs in patients with cancer (cancer-related anemia, CRA), either as a tumor-driven blood disorder or as a consequence of a patient’s chemotherapy or progressive disease (Madeddu et al., 2018). The prevalence of CRA is notably high and varies according to the type and stage of cancer (Macciò et al., 2015). Although CRA is usually considered as a chemotherapy side effect, more than 30% of cancer patients are anemic at the time of diagnosis, before the initiation of any chemotherapy regimen (Ludwig et al., 2004). In accordance, cancer cells can directly cause anemia by suppressing erythropoiesis through either bone marrow infiltration or cytokine-mediated changes in iron availability. In cancer patients, anemia may also result from underlying comorbid conditions such as hemolysis, coagulation disorders, nutritional deficiencies, and renal insufficiency. (Gaspar et al., 2015). Furthermore, the antineoplastic treatment itself may be a major cause of anemia, or alternatively, may exacerbate the pre-existing anemia in these patients (Tas et al., 2002). Regardless of the underlying cause, anemia complicates the course of cancer and negatively influences the therapeutic outcome (Clarke and Pallister, 2005).

Current treatment options for CRA include erythropoietin-stimulating agents (ESA), iron therapy, and red blood cell (RBC) transfusions. The transfusion of RBC rapidly increases hemoglobin concentration and improves functional status in cancer patients (St Lezin et al., 2019). Despite these benefits, several adverse reactions may occur during or after RBC transfusions, negatively affecting patient outcomes (Goubran et al., 2016). The use of ESA, which acts as erythropoietin receptor (EPOR) agonists, reduces the need for RBC transfusions and improves the quality of life in patients with CRA. However, a growing body of evidence demonstrates that, when using ESA for treating anemia outside chemotherapy and aiming at near-to-normal hemoglobin levels, ESA therapy increases the risk of cancer progression or recurrence, as well as the risks of thromboembolic events and arterial hypertension in these patients (Hedley et al., 2011; René et al., 2017; Horváth-Puhó et al., 2018). In addition, approximately 20% of ESA users are non-responders, exhibiting neither a reduction nor a significant change in hemoglobin levels within the first 3 months of ESA treatment (Ingrasciotta et al., 2016). Hence, ESA reduces the risk for RBC transfusion but increases the risk for thromboembolism, leading guidelines to recommend their use only in very specific instances, taking into consideration whether complete recovery is an anticipated outcome. Iron replacement therapy may be additionally used to improve hemoglobin response and reduce the need for RBC transfusions in patients with chemotherapy-associated anemia receiving ESA (Bohlius et al., 2019). Intravenous iron is recommended for the treatment of absolute iron deficiency anemia with ferritin levels below 30 ng/ml and transferrin saturations of less than 20% (Gilreath and Rodgers, 2020). Nevertheless, due to iron toxicity and its possible association with the treatment of cancer, iron replacement therapy could not be recommended for patients with functional iron deficiency and high levels of ferritin (Puliyel et al., 2015; Madeddu et al., 2018). Bearing in mind the above-mentioned therapeutic limitations, despite its clinical significance in cancer patients, CRA still remains an underestimated and therefore largely untreated chronic condition.

**FIGURE 1:**
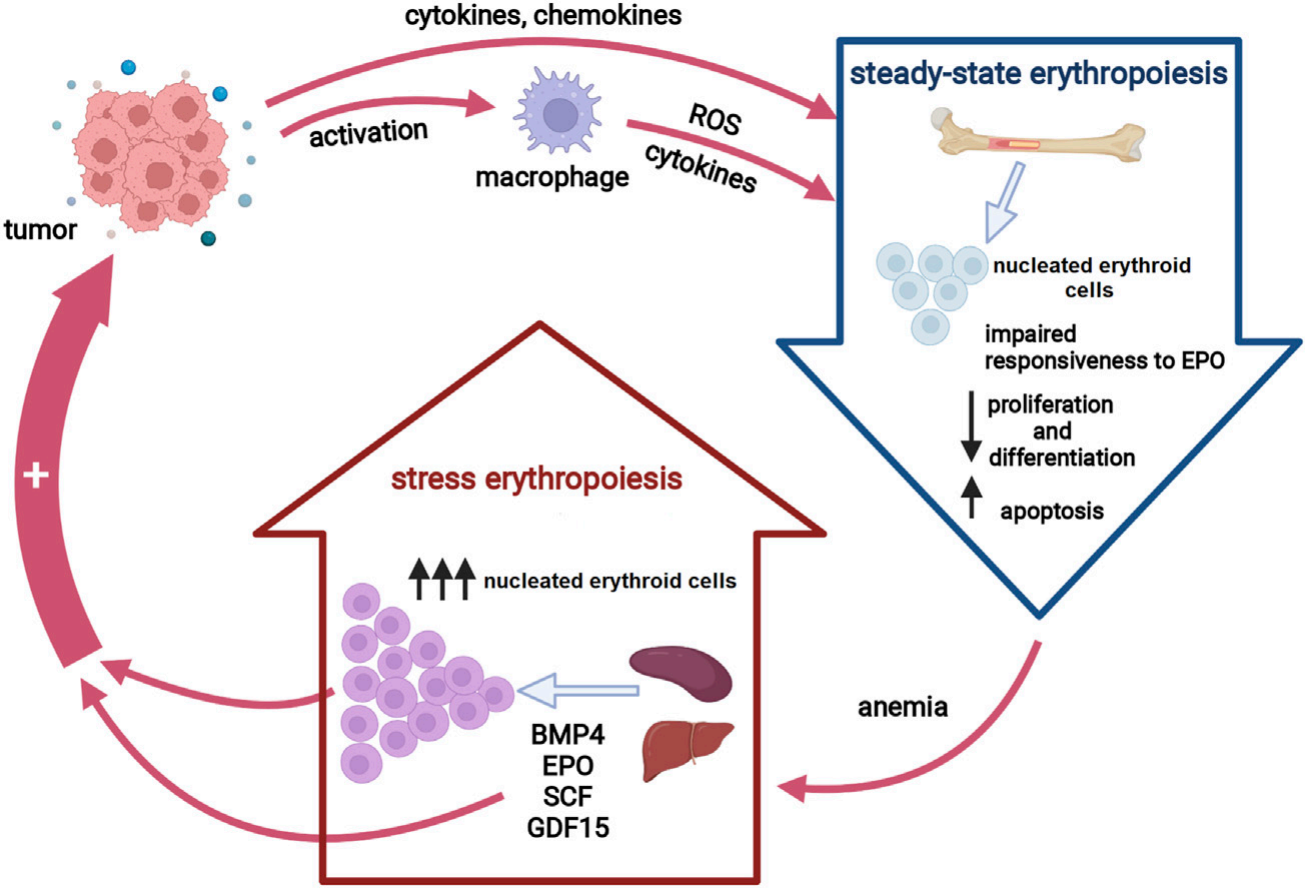
Erythropoiesis in cancer. Tumor-induced inflammatory cytokines suppress erythropoiesis in the bone marrow either directly or via macrophage activation, resulting in decreased red blood cells production. Anemia activates stress erythropoiesis pathways at extramedullary sites to provide rapid expansion of nucleated erythroid cells. Both activated stress erythropoiesis pathways and nucleated erythroid cells may promote tumor progression. Abbreviations: EPO, erythropoietin, ROS, reactive oxygen species; BMP4, bone morphogenic protein 4; SCF, stem cell factor; GDF15, growth differentiation factor 15. Created with BioRender.com.

Similar to anemia of other chronic conditions, CRA is mostly referred to as the anemia of inflammation (Weiss and Goodnough, 2005). Several lines of evidence point toward pro-inflammatory cytokines as key players in the pathogenesis of CRA (Maccio et al., 2015). Thus, tumor-driven overexpression of certain inflammatory cytokines results in an impaired erythropoietin (EPO) production, an inadequate response of the erythroid progenitors to EPO as well as an altered iron metabolism (Cullis, 2011). In addition, pro-inflammatory cytokines exert toxic effects on erythroid progenitor cells by inducing the formation of free radicals, predominantly by neighboring macrophages (Weiss and Goodnough, 2005). Macrophage-derived cytokines may also contribute to reduced iron availability by increasing hepatic hepcidin synthesis. Hence, steady-state erythropoiesis under chronic inflammatory conditions is insufficient to compensate for the anemia due to two main mechanisms: iron restriction and direct action of pro-inflammatory cytokines on erythroid progenitors (Natalucci et al., 2021). In order to maintain erythroid homeostasis in chronic inflammatory states, inflammatory signals induce a process termed stress erythropoiesis (SE) (Bennett et al., 2019). Activation of SE pathways ensures an extensive expansion of a distinct population of immature progenitor cells, so-called stress erythroid progenitors, under increased demands for RBC. However, chronic SE causes an imbalance in erythroid proliferation and differentiation, leading to a net increase in the number of immature erythroid cells, and subsequently exacerbates the anemic condition. Apart from aggravating CRA, the activation of SE pathways may reshape the tumor microenvironment to further support cancer growth (Martínez et al., 2017; Foster et al., 2018) (Figure 1). Unraveling the roles of SE signaling components and stress erythroid progenitors as well as the mechanisms underlying impaired erythropoiesis in cancer patients are the essential steps toward establishing an optimal and personalized CRA treatment approach, and therefore, a promising strategy to reduce the tumor-promoting activity of CRA-initiated SE pathways.

Bone morphogenic protein 4 (BMP4), EPO, stem cell factor (SCF) and growth/differentiation factor-15 (GDF15) have emerged as the key players in SE. Apart from regulating SE, these factors also play roles in erythroid development during fetal and/or adult period. Thus, BMP4 is essential for the expansion of erythroid progenitors during fetal development. Unlike bone marrow progenitors, fetal liver progenitor cells can give rise to early erythroid progenitors in the presence of EPO alone, without any additional signals, reflecting the differences between adult bone marrow and fetal liver erythropoiesis (Porayette and Paulson, 2008). The expression levels of BMP4 correlates with the level of erythropoietic activity in the fetal liver, pointing towards BMP4 as an essential signal to maintain the erythroid homeostasis and support the rapid growth of the fetus. Similar to fetal liver, in response to anemia, BMP4-dependent signaling, which is regulated by hypoxia, initiates the rapid and extensive expansion of erythroid progenitors in the adult spleen (Lenox et al., 2005). Accumulating evidence now suggests that, although GDF15 is not essential for steady-state RBC production, it appears to be critical for maintaining the hypoxia-dependent expression of BMP4 during SE (Hao et al., 2019). It was further demonstrated that hypoxia, BMP4 and SCF cooperatively regulate the expansion of splenic early erythroid progenitors in response to anemia (Perry et al., 2007). SCF is required for the maintenance of erythroid progenitor cells under both steady-state and stress conditions. SCF preserves the undifferentiated state of erythroid progenitors and sustained signaling by the SCF leads to an expansion of immature erythroid cells. In addition, SCF negatively affects terminal erythroblast maturation through the crosstalk with the signaling induced by EPO (Haas et al., 2015). EPOR signaling is necessary for the survival of late-stage erythroid progenitors in the fetal liver and adult bone marrow. Hypoxia induces EPO expression in the fetal liver, adult liver and adult kidney. This is a vital response for the regulation of erythropoiesis under conditions of anemia or chronic hypoxia. EPOR is expressed at low levels on early erythroid progenitor cells and then upregulated during erythroid differentiation until late erythropoiesis when EPO is no longer required for erythroid cell survival (Noguchi et al., 2008; Hattangadi et al., 2011).

## Evolving Concepts of Stress Erythropoiesis

Erythropoiesis is a well-orchestrated and tightly regulated process for maintaining a steady number of RBC and thus providing adequate oxygen delivery to tissues. Since RBC are critically important for survival, erythropoiesis during development occurs in two distinct forms. The first is termed “primitive” form and refers to nucleated erythroid cells that originate extraembryonically in the yolk sac. Apart from being essential for the oxygen delivery to cells within the rapidly growing embryo, these large nucleated erythroid cells also seem to play an important role in angiogenesis (Baron, 2013). The second wave of erythropoiesis -“definitive” form consists of enucleated erythroid cells that derive predominantly from the fetal liver and spleen (Palis and Segel, 1998).

In contrast to the embryonic or fetal period, erythropoiesis in healthy adults is primarily homeostatic and occurs in the bone marrow. Under steady-state conditions, RBC are formed continuously and mature within the specialized microenvironmental niches, known as erythroblastic islands (Socolovsky, 2013; de Back et al., 2014). The sequential differentiation of multipotent hematopoietic stem cells into fully mature RBC is a multistep process that involves distinct populations of erythroid committed cells, starting from the earliest burst forming erythroid (BFU-E). These earliest committed erythroid progenitor cells are capable of self-renewal and ultimately differentiate into the colony-forming units erythroid (CFU-E). Subsequent maturation of CFU-E results in a cascade of morphologically recognizable erythroid precursors, and finally reticulocytes that are released into the circulation (Dzierzak and Philipsen, 2013).

Steady-state erythropoiesis is critically regulated by the kidney-derived hormone—EPO. EPO promotes the survival, proliferation and differentiation of erythroid progenitors, primarily CFU-E cells. Binding of EPO to the EPOR on the surface of erythroid cells upregulates the expression of its own receptor, which increases EPO sensitivity as well as the expression of erythroid-specific transcription factors, such as GATA1 and KLF1, and other erythroid-specific genes (glycophorin and globin genes). In addition to EPO, BFU-E cells respond to many cytokines and growth factors including interleukin-3, SCF, insulin like growth factor-1 and glucocorticoids (Hattangadi et al., 2011). During erythropoietic development, direct cell–cell contacts provide the necessary cues for erythroid cell maturation. Thus, direct contact of erythroblasts with macrophages is required for enhanced erythroblast proliferation that results from decreased transit time in the G0/G1 phase of cell cycle (Rhodes et al., 2008). Furthermore, the erythroblast macrophage protein, which is expressed on both erythroblasts and macrophages, is substantial for terminal maturation of erythroblasts. In addition to direct cellular contact, cells within erythroblast island secrete different soluble factors that regulate the rate of erythropoiesis via positive and negative feedback mechanisms. Differentiating erythroblasts secrete angiogenic factors including vascular endothelial growth factor A and placenta growth factor that promote their interactions either with macrophages in erythroblastic islands or with endothelial cells and therefore facilitate the passage of erythroid cells through the endothelial barrier. On the other hand, during chronic inflammation, macrophages within erythroblast island may secrete the proinflammatory cytokines such as interleukin-6, tumor necrosis factor-α and interferon-γ, that suppress erythropoiesis in various ways (Chasis and Mohandas, 2008).

Of paramount importance for erythropoiesis is to adjust the rate of RBC production to meet physiological demands. Hence, steady-state erythropoiesis generates new RBC at a constant rate, sufficient to replace the ones that are being destroyed. However, there are times of great erythropoietic need, caused either by prolonged or severe anemia, along the time when steady-state erythropoiesis is impaired or inhibited. At these times, homeostatic erythropoiesis in the bone marrow is not sufficient to support adequate RBC production, and therefore mechanisms of SE are initiated (Lenox et al., 2005).

The current understanding of SE relies mainly on data gathered from mouse studies and experimental data have demonstrated that SE utilizes progenitor cells and signals distinct from those in bone marrow during steady-state erythropoiesis. The regulation of erythropoiesis is largely dependent on microenvironmental cues, and unlike steady-state erythropoiesis in the bone marrow, SE occurs predominantly in the mouse spleen (Paulson et al., 2011). The process referred to as SE was initially observed and analyzed in the context of experimentally-induced acute anemia or bone marrow transplantation (Dornfest et al., 1992; Nibley and Spangrude, 1998; Paulson et al., 2011). Thus, the dynamics of BFU-E and CFU-E in the bone marrow, peripheral blood, and spleen were significantly altered following phenylhydrazine-induced anemia or allogeneic bone marrow transplantation (Hara and Ogawa, 1976; Vannucchi et al., 1995). The presence of erythroid progenitors in the peripheral circulation, along with a marked increase in splenic BFU-E cell number of phenylhydrazine-treated mice have suggested that these early erythroid cells migrate from the bone marrow to the spleen in response to acute anemia. Spleen microenvironment has been subsequently shown to be superior in promoting the rapid expansion of erythroid progenitors during acute anemia (Perry et al., 2007; Harandi et al., 2010). Moreover, the same phenomena of erythroid progenitor cell migration and extensive erythroid self-renewal have also been observed afterward in the setting of chronic anemia caused by inflammation (Millot et al., 2010; Bennett et al., 2019), stress (Vignjević et al., 2014; McKim et al., 2018), hematological disorders (Crielaard and Rivella, 2014), and cancer (Liu et al., 2015; Sano et al., 2021).

Erythroid progenitors are decisive in determining the overall red cell mass and the findings derived from mouse studies have revealed that the characteristics of these cells, as well as the mechanisms that drive their extensive expansion during SE in many ways differ from steady state erythropoiesis and resemble those involved in erythropoiesis during embryonic and fetal development (Porayette and Paulson, 2008; Alter et al., 2013; Xiang et al., 2015). It is now widely accepted that fetal and adult hematopoietic stem/progenitor cells have distinct self-renewal mechanisms and lineage outputs (Chen et al., 2019). Low levels of oxygen that occur naturally in developing embryos and fetuses, resulting in the activation of a physiological stress response designed to support rapid blood formation (Simon and Keith, 2008). This physiological response requires a special population of embryonic/fetal erythroid progenitors that have a great ability to generate large numbers of RBC. The extensive proliferation of developmental erythroid progenitors is a key component of this response, which is highly supported by the microenvironments of the fetal liver and spleen. Similarly, in response to anemic stress, the adult mouse spleen and liver provide a suitable niche for rapid expansion of a distinct population of stress erythroid progenitors, designated as “stress BFU-Es” (Lenox et al., 2009; Paulson et al., 2011). These stress erythroid progenitors have a much greater self-renewal capacity compared to bone marrow steady-state progenitors and the mechanisms that regulate their expansion depend primarily on signals derived from the spleen microenvironment (Harandi et al., 2010).

Animal models have been crucial to the advances in understanding the mechanisms of SE activation and regulation (Lenox et al., 2005; Millot et al., 2010; Ulyanova et al., 2016; Vignjević Petrinović et al., 2020). Accumulating data from different mouse models suggest that, unlike steady-state erythropoiesis, Hedgehog and BMP4 signaling along with glucocorticoids, EPO, SCF, GDF15 and hypoxia-inducible factors plays a pivotal role in SE (Perry et al., 2009; Millot et al., 2010; Voorhees et al., 2013; Vignjević et al., 2014; Hao et al., 2019; Wang et al., 2021). The activation of the glucocorticoid receptor is essential for the expansion of immature erythroid cells during SE (Bauer et al., 1999; Vignjevic et al., 2015) and it seems that glucocorticoids act synergistically with hypoxia-inducible factor-1 alpha (HIF-1α)and GDF15 to promote rapid proliferation of stress erythroid progenitors whose self-renewal is critically dependent on BMP4 signaling (Flygare et al., 2011; Hao et al., 2019). Hypoxia pathway proteins play both direct and indirect roles during SE. Each functional HIF unit is a heterodimer consisting of an oxygen-sensitive alpha-subunit (HIF-1α, 2α or 3α) and a constitutively expressed beta-subunit (HIF-β). Wu and Paulson (2010) have demonstrated that stress-induced BMP4 expression in the spleen is regulated at the transcriptional level by HIF. In particular, up-regulation of BMP4 expression, primarily regulated by HIF-2α, has been defined as a key signal involved in SE, and a putative HIF-binding site has been identified at the 3′ end of the *BMP4* gene (Lenox et al., 2005). However, several observations suggested that HIF-dependent regulation of BMP4 during SE may be more complex (Wu and Paulson, 2010). The results obtained from studies examining HIF-dependent transcription of BMP4 in hepatocellular carcinoma cells have revealed an indirect role for HIF-1α in the regulation of BMP4 expression (Maegdefrau et al., 2009). Furthermore, during SE, the expression of BMP4 is delayed in the spleens of flexed-tail (f/f) mutant mice, which have a mutant SMAD5 with the ability to also inhibit the function of SMAD1 and 8, suggesting that SMAD1/5/8 may act concordantly with HIF-2α to regulate transcription of BMP4 in the spleen under SE conditions. Additionally, new evidence suggests that HIF-3α might be involved in SE as well (Wang et al., 2021).

Apart from these key SE mechanisms, a variety of additional microenvironmental signals that derive from spleen or liver are required for optimal erythroid stress response in mice (Vignjević Petrinović et al., 2016; Liao et al., 2018; Trivedi et al., 2019; Ulyanova et al., 2020). Nevertheless, *in vitro* culture of human bone marrow has revealed an analog population of human bone marrow cells that can also differentiate into BMP4-dependent stress erythroid progenitors under SE conditions (Xiang et al., 2015). In accordance, analysis of human SE in patients with congenital anemias or following bone marrow transplant (Meletis et al., 2001; Alter et al., 2013) points toward SE-related pathways as a highly conserved mechanism in response to anemia. Moreover, emerging evidence demonstrates that severe COVID19 patients suffer from anemia and show a significant increase in circulating erythroid progenitor cells, suggesting that SARS-CoV-2 infection induces SE as well (Huerga Encabo et al., 2021).

## Nucleated Erythroid Cells: New Players in Cancer Progression

An immune response to infection could interfere with anti-tumor immunity through interactions between immune pathways involved in protection against infectious agents and cancer cells (Jacqueline et al., 2017). In addition, persistent infections have the potential to induce chronic inflammatory states. Chronic inflammation promotes tumor progression and treatment resistance *via* multiple mechanisms. The proinflammatory mediators orchestrate cross-talk between cancer cells and their microenvironment to create a tumor-supporting immunosuppressive niche, thereby counteracting immune surveillance and facilitating the survival of transformed cells (Wang and DuBois, 2015; Trivanović et al., 2020). Among the immune-associated cells in the tumor microenvironment, macrophages and myeloid-derived suppressor cells play the most prominent immunosuppressive roles (Fang et al., 2017; Dou and Fang, 2021). Furthermore, recent studies have brought a renewed focus on the immunomodulatory role of nucleated RBC (Elahi et al., 2020; Kanemasa et al., 2021). Apart from the retention of intact nuclei, these erythroid cells display molecular features of an immature erythroid phenotype, such as high expression of the transferrin receptor protein 1 (CD71), and also express genes encoding immune checkpoint molecules, such as CD274 (encoding PD-L1) and CD244 (encoding 2B4) (Sano et al., 2021).

The association between erythropoiesis and immunomodulation was first reported in the late 1970s (Conway de Macario and Macario, 1979). Initial findings showed an immunosuppressive activity of nucleated RBC from both neonatal and adult murine spleen (Pavia and Stites, 1979; Macario et al., 1981). Later studies have demonstrated that immature RBC from human bone marrow and peripheral blood display a wide range of immunomodulatory properties, as well (Sennikov et al., 2004; Kanemasa et al., 2021). Certainly, CD71-expressing erythroid cells contribute to fetomaternal tolerance and new evidence suggests that their depletion during fetal development leads to the failure of gestation due to the immunological rejection of the fetus (Delyea et al., 2018). Less clear, however, is the physiological significance of the immunosuppressive properties of immature erythroid cells in adulthood, as well as their pathological implications. Furthermore, the other outstanding question is the precise characterization of nucleated erythroid cells exhibiting immunomodulatory properties. According to the literature, these cells represent a heterogeneous population of nucleated erythroid cells predominantly expressing CD71 antigen (Elahi, 2014; Namdar et al., 2017; Kanemasa et al., 2021). A subpopulation of these CD71^+^ erythroid cells co-express CD235a in humans and Ter119 in mice, respectively (Elahi, 2019). In addition, some of these CD71^+^ erythroid cells also express a pan-leukocyte marker CD45, that is, lost during erythroid differentiation process and therefore may be used as a marker of early-stage nucleated erythroid cells (Grzywa et al., 2021). Several studies have shown that CD71^+^ erythroid cells modulate the immune response against virus infection, such as human immunodeficiency virus, Hantan virus, respiratory syncytial virus, and SARS-CoV-2 (Namdar et al., 2019; Rinchai et al., 2020; Huerga Encabo et al., 2021; Zhang et al., 2021). Moreover, the abundance of these cells in the peripheral blood of virus-infected patients has been associated with the clinical severity of infection.

**FIGURE 2:**
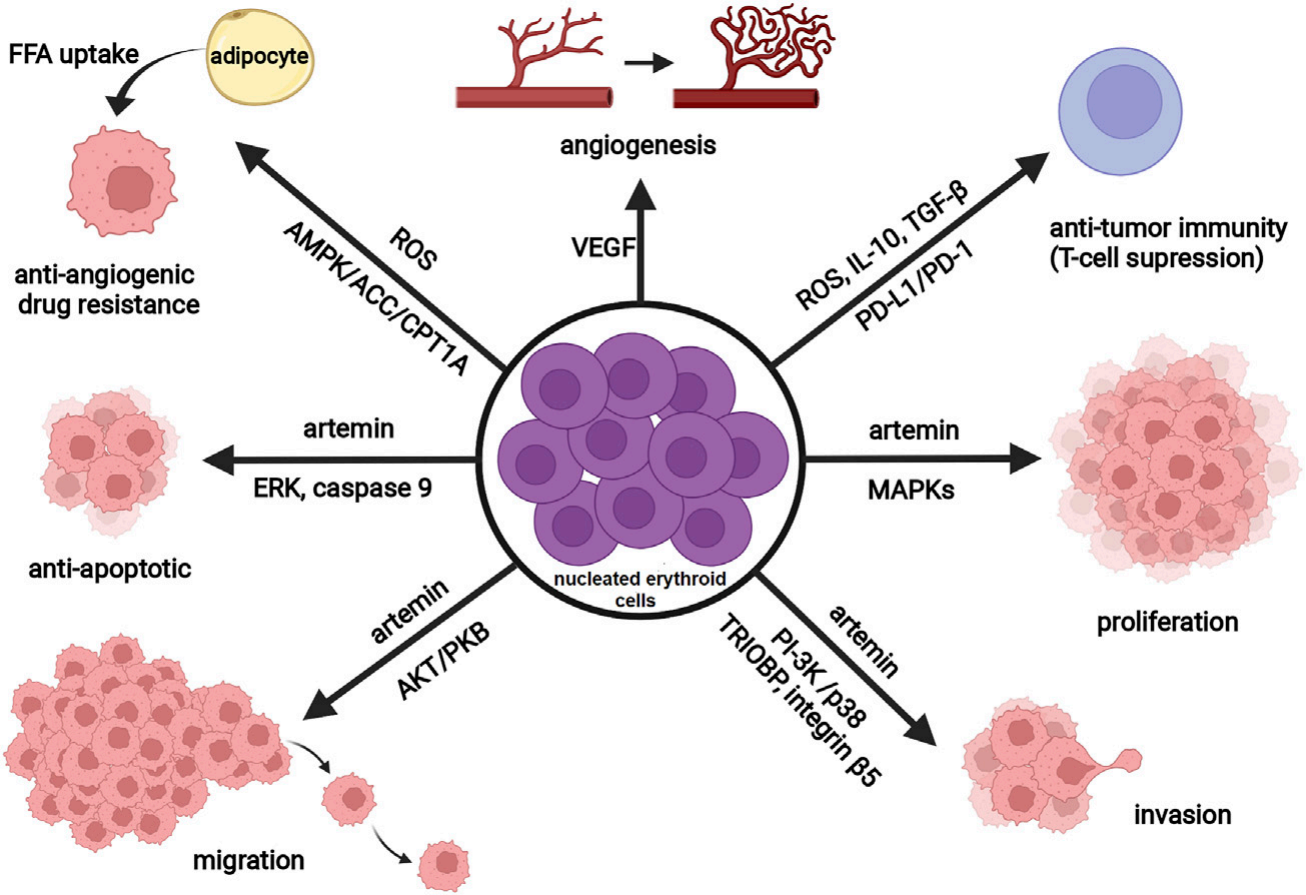
Schematic representation of the tumor-promoting activity of nucleated erythroid cells. Nucleated erythroid cells promote tumor angiogenesis (vascular growth) through the secretion of vascular endothelial growth factor (VEGF). By producing reactive oxygen species (ROS), transforming growth factor (TGF-β), interleukin 10 (IL-10), and expressing programmed death-ligand 1 (PD-L1), nucleated erythroid cells decrease anti-tumor immune response (T-cell suppression). Released ROS also contribute to antiangiogenic drug resistance via regulating lipid metabolism (through AMP-activated protein kinase (AMPK)-acetyl CoA carboxylase (ACC)-carnitine palmitoyl transferase 1 (CPT1A) pathway) and induce cancer-associated adipocytes to feed tumor with free fatty acids (FFA). By releasing a neurotrophic peptide—artemin, nucleated erythroid cells promote tumor proliferation (MAPK signaling pathway), invasion (stimulate expression of TRIOBP and integrin beta5 through PI-3K and p38 signaling pathway), migration (Akt/PKB signaling pathway), and survival (anti-apoptotic effect through ERK activation and phosphorylation of caspase-9). Abbreviations: MAPK, mitogen activated protein kinase; TRIOBP, TRIO and F-actin Binding Protein; PI-3K, phosphatidylinositol 3-kinase; PKB, protein kinase B; ERK, extracellular signal-regulated kinase. Created with BioRender.com.

In recent years there has been growing research interest in studying the interactions among CD71^+^ erythroid cells, immune cells, and tumor cells, as well as their implications for tumor progression (Han et al., 2018; Chen et al., 2021). As noted previously, some CD71^+^ erythroid cells co-express CD45 antigen (Grzywa et al., 2021). This CD45^+^ erythroid cell subpopulation is highly abundant in tumor-bearing mice and seems to be primarily responsible for the immunosuppressive activity of erythroid progenitor cells (Zhao et al., 2018). Experimental data have shown that tumors trigger renal production of EPO and EPO-dependent expansion of immature erythroid cells (Xue et al., 2011; Sano et al., 2021). These erythroid cells expand rapidly at extramedullary sites, such as the spleen (Han et al., 2018; Zhao et al., 2018), and subsequently infiltrate the tumor microenvironment, as recently detected in hepatocellular carcinoma and glioblastoma (Chen et al., 2021; Grzywa et al., 2021; Lu et al., 2021). Alternatively, erythroid cells, like other hematopoietic cells within the tumor microenvironment, may originate from cancer stem cells (Hassan and Seno, 2020), and accumulating evidence now supports this novel hypothesis (Zhang et al., 2013; Yang et al., 2018). Regardless of their origin, nucleated erythroid cells dynamically interact with cancer cells, immune cells, and stromal cells, thus shaping the tumor microenvironment. Through these interactions, erythroid cells promote tumorigenesis by enhancing the survival, migration, and proliferation of cancer cells and/or suppressing T cell-mediated antitumor immunity (Figure 2). Namely, Han et al. (2018) reported that tumor-induced nucleated erythroid cells promote hepatocellular carcinoma progression by releasing a neurotrophic peptide - artemin that binds to its receptor on cancer cells. In accordance, Li et al. (2021) found a significantly increased number of artemin-expressing splenic erythroid cells in patients with pancreatic ductal adenocarcinoma and point toward nucleated erythroid cell count as an independent predictor of poor outcome in these patients. Moreover, Hou et al. (2021) have recently demonstrated that both radiotherapy and immunologic checkpoint blockade with antibodies against the anti–programmed death ligand 1 (PD-L1) deplete tumor-induced erythroid progenitors in the spleen and subsequently reduce artemin secretion and tumor progression. The transmembrane protein PD-L1 is highly expressed on tumor-infiltrating erythroid progenitor cells (Sano et al., 2021), making them a promising target for cancer immunotherapy.

In addition to effects on antitumor immunity and cancer cells, nucleated erythroid cells may contribute to tumor angiogenesis (Tordjman et al., 2001) and antiangiogenic drug resistance (Xia et al., 2020). Markedly increased number of CD45^+^ erythroid cells has been found in patients with diffuse large B cell lymphoma presenting with anemia. Moreover, using the animal model of diffuse large B cell lymphoma, Xia et al. (2020) have found that these erythroid cells produce reactive oxygen species in a hypoxic microenvironment, thereby influencing lipid metabolism in tumor-surrounding adipocytes and subsequently contribute to antiangiogenic drug resistance. Bearing in mind that EPOR-expressing erythroid cells are the main target cells for EPO, these findings are in accordance with previously published data implying that adequate inhibition of EPO function in cancer patients might improve the therapeutic efficacy of antiangiogenic therapy (Nakamura et al., 2017).

## Bone Morphogenetic Protein 4/BMPR Signaling in Cancer

Bone morphogenetic proteins (BMPs) are signaling molecules well-known for their essential roles during vertebrate development. The BMP family belongs to the transforming growth factor *β* (TGF-β) superfamily and contains over 20 members with a wide variety of functions (Zhang and Li, 2005). Based on sequence homology, the BMP family members can be further classified into several subgroups, including the BMP2/4 subgroup of structurally similar proteins (Katagiri and Watabe, 2016). The action of BMPs is mediated through both canonical and non-canonical signaling pathways. The canonical signaling is initiated upon BMPs binding to heterotetrameric receptor complexes composed of two types I and two types II serine/threonine kinase receptors (BMPRI and BMPRII), which further activate the intracellular SMAD and non-SMAD pathways (Wang et al., 2014). In the presence of BMP4, BMPRIA and BMPRIB recruit BMPRII and initiate signaling via phosphorylation of SMAD1/5/8. Thus, BMP4/SMAD5 dependent signaling is required for the expansion of erythroid progenitors during fetal development and SE in adults (Porayette and Paulson, 2008; Lenox et al., 2009). Besides canonical signaling, BMP4 was also found to activate SMAD-independent p38 mitogen-activated protein kinase pathway, exerting pro-angiogenic effects (Zhou et al., 2007; García de Vinuesa et al., 2016).

The essential role of BMP4 in erythroid cell development (Porayette and Paulson, 2008) and its ability to regulate vital processes, such as angiogenesis and hematopoiesis (Goldman et al., 2009; Ciais and Bailly, 2012) have made this signaling molecule an increasingly interesting topic in cancer research (Kallioniemi, 2012). So far, BMP4 has been directly implicated in the pathogenesis of pituitary prolactinoma and colorectal cancer (Paez-Pereda et al., 2003; Tomlinson et al., 2011). The expression levels of BMP4 have been extensively studied in various cancer cell lines and tumor tissues of human and animal origin, by a variety of methods. This protein was detected in both normal and tumor tissues at variable levels (Rothhammer et al., 2005; Deng et al., 2007; Goulley et al., 2007; Yang et al., 2021). Comparative immunohistochemical analysis of BMP4 expression in different tumors and corresponding normal tissues (Alarmo et al., 2013) has revealed a strong granular BMP4 staining pattern in several normal human tissues (e.g., stomach, esophagus, liver, and spleen), indicating a significant function of BMP4 in tissue homeostasis. Different patterns of BMP4 protein expression were shown in multiple tumor samples, with exceptionally strong granular BMP4 immunostaining observed in tissue samples of squamous cell carcinomas. The results of Alarmo et al. (2013) also demonstrated that strong BMP4 expression in breast cancer tissue samples was associated with a low rate of tumor cell proliferation as well as with an increase in tumor recurrence, reflecting its dual role in breast cancer. Thus, a growing body of evidence suggests that BMP4 exerts its effects on breast cancer cells in two opposite directions—acting as a suppressor of primary tumor growth and simultaneously promoting cancer cell migration and invasion (Ketolainen et al., 2010; Choi et al., 2019). In addition to breast cancer, BMP4 promotes tumor progression, angiogenesis, and metastasis in multiple cancer types (Rothhammer et al., 2007; Maegdefrau et al., 2009; Martínez et al., 2017; Zhou et al., 2018; Ju et al., 2019; Deng et al., 2020), but opposite and conflicting results have also been reported (Cao et al., 2014; Zhao et al., 2019). Furthermore, in some tumor types, such as pituitary adenomas, BMP4 plays cell type-specific roles—it stimulates pituitary prolactinoma while inhibiting corticotroph tumor cells (Labeur et al., 2010). Thus, in general, the consequences of BMP4 activation in the tumor microenvironment are likely dependent on cellular composition and the context-specific BMP4 signaling within a particular cell type. Moreover, BMP4 often forms heterodimers with BMP7 that are more active than homodimers (Neugebauer et al., 2015), and therefore the effects of BMP4/BMPR activation *in vivo* settings do not necessarily reflect just BMP4 activity. Additional studies on the BMP4/BMPR signaling pathway in a large number of different primary cells/cell lines from each tumor type are further needed to clarify the exact contribution of BMP4 signaling to tumorigenesis and metastasis in a tissue-specific manner (Schneider et al., 2017). Identification of distinct mechanisms underlying the context-specific BMP4 interactions within the tumor microenvironment could provide therapeutic targeting of the BMP4 pathway according to specific tumor phenotypes.

## Erythropoietin/Erythropoietin Receptor Axis in Tumor Progression

Apart from playing an indispensable role in the regulation of erythropoiesis, EPO exerts pleiotropic effects in a wide range of tissues (Lombardero et al., 2011). The binding of EPO with EPOR homodimer induces a conformational change that causes subsequent activation of various kinases and downstream pathways including JAK2-STAT5, RAS-RAF-MAPK, PI3K-AKT, and protein kinase C pathway (Kuhrt and Wojchowski, 2015). Among them, JAK2-STAT5 and RAS-RAF-MAPK pathways are usually related to EPO mitogenic effects, while the PI3K-AKT signaling is commonly associated with its anti-apoptotic activities. Recently, new EPO/EPOR signals and target molecules have been identified (Tóthová et al., 2021). These molecules are predominantly involved in the processes of epigenetic regulation, mRNA splicing, EPOR turnover, and negative regulation of STAT5 signaling, but some of them are previously unexpected EPO target molecules, such as erythroid cytoskeletal targets (spectrins, adducin 2, glycophorin C) or metabolic regulators (aldolase A, pyruvate dehydrogenase α1, thioredoxin-interacting protein) (Held et al., 2020).

Production of EPO is regulated at the transcription level and increases mainly in response to hypoxia or anemia by the hypoxia-inducible transcription factors (Jelkmann, 2011). Hypoxia-inducible EPO signaling has been suggested to play a significant role in cancer’s aggressive behavior and drug resistance (Acs et al., 2003; Miyake et al., 2013). Although EPORs expression has been demonstrated in different tumor tissues (Kumar et al., 2017), the critical issues regarding the specificity of the commercially available anti-EPOR antibodies for immunohistochemistry have been raised and therefore a consensus on both the expression and potential functional significance of EPORs in cancer cells has not been reached so far (Bennett et al., 2016). Several studies showed that many investigations relying on detection of the EPOR were not reproducible because of the antibody issues and pointed towards the widespread use of nonspecific anti-EPOR antibodies that provide false positive staining with tissues as a main reason for the conflicting data on EPOR protein expression (Elliott et al., 2012; Elliott et al., 2013). In accordance, using a rabbit monoclonal antibody specific to human EPOR as well as a western blot technique, Elliott et al. (2013) have reported that EPOR are not detectable in primary human tumor tissue samples.

The activation of the main EPO/EPOR downstream pathways (JAK2-STAT5, PI3K-AKT, MAPK/ERK) has been demonstrated in cancer cells (Paes and Ringel, 2008; Thomas et al., 2015; Guo et al., 2020). Several lines of evidence suggest that EPOR downstream signaling in cancer cells may exhibit distinct features as compared to erythroid progenitor cells (Tóthová et al., 2021). For example, tumor cells has been shown to express considerably lower levels of EPOR than erythroid progenitor cells (Swift et al., 2010), and aside from the “classical” EPOR homodimer form, usually detected on erythroid cells, either a heteroreceptor consisting of EPOR and common beta receptor (EPOR/βcR) or Ephrin B4 receptor (EPHB4) has also been identified as EPO-binding receptor in cancer cells (Arcasoy et al., 2002; Pradeep et al., 2015). The membrane-bound ephrin-B2 ligand and its receptor EPHB4 are expressed in tumor-specific blood vessels and appear to play roles in tumor-associated angiogenesis. The recombinant human erythropoietin competes with ephrin-B2 for binding to EPHB4, and therefore, the tumor progression might be mediated via EPO-induced phosphorylation of EPHB4 and subsequent activation of downstream signaling, independent of the EPOR (Gilreath and Rodgers, 2020). Furthermore, some cancer cells may exhibit constitutive activation of EPOR pathways (Fu et al., 2009), suggesting that observed effects of signal transduction through the EPOR in these cells are EPO-independent. In addition to EPOR homodimer form, heterodimeric EPOR/βcR form has also be demonstrated in macrophages (Nairz et al., 2011; Brines and Cerami, 2012). After binding to its specific receptor on macrophages, EPO exerts anti-inflammatory effects directly by inhibiting pro-inflammatory immune effector pathways (Nairz et al., 2011) or indirectly by increasing macrophage-mediated T cell suppression (Wood et al., 2016). In this way, EPO may activate tumor-associated macrophages in the tumor microenvironment, thereby suppressing the antitumor immune response and subsequently leading to cancer progression (Li et al., 2020). Alternatively, EPO may affect vascular endothelial cells and promote tumor angiogenesis (Hardee et al., 2007; Tankiewicz-Kwedlo et al., 2016). In accordance, Nakamura et al., (2017) have shown that vascular endothelial cells are the primary target of EPO protein in the tumor microenvironment. Alongside its influence on the tumor microenvironment, EPO might also have a direct effect on cancer stem cells by increasing the proliferation and self-renewal of these cells (Cao et al., 2010). Likewise, Todaro et al. (2013) have demonstrated that breast cancer stem-like cells isolated from tumor tissues express the EPOR and respond to EPO treatment with increased proliferation and resistance to chemotherapeutic agents. The role of EPOR in both cancer-initiating cell self-renewal and the resistance of breast cancer cells to treatment has been confirmed in subsequent studies (Zhou et al., 2014; Zsóková et al., 2019). Interestingly, EPOR knockdown experiments showed that reduced EPOR expression in estrogen receptor-positive breast cancer cells resulted in decreased proliferation of these cancer cells, but the same effect has not been observed in estrogen receptor-negative breast cancer cells, indicating that EPOR signaling is strongly dependent on breast cancer biology (Reinbothe et al., 2014). However, the limitations regarding the specificity of used EPOR antibodies should always be considered in the interpretation of the above-mentioned results.

Taken together, due to the widespread use of nonspecific anti-EPOR antibodies that may provide false positive staining, the tumor-promoting effects of the EPO/EPOR axis have remained an outstanding issue. Further studies are needed to address this issue. In addition to antibody issues, the specific tumor biology should always be carefully considered in the interpretation of these results.

## Targeting the Stem Cell Factor/C-Kit Interaction in Cancer

The transmembrane tyrosine kinase receptor CD117, commonly known as c-Kit, is encoded by a proto-oncogene c-kit that was initially identified as a homolog of the feline sarcoma viral oncogene v-kit (Besmer et al., 1986). SCF is a cytokine that binds to the c-kit receptor and triggers signaling through receptor dimerization and intermolecular tyrosine phosphorylation. Downstream of c-Kit, several signal transduction pathways are activated: PI3-K, Src family members, JAK-STAT, and RAS-RAF-MAPK pathways that play a critical role in hematopoiesis, pigmentation, reproduction as well as in gut function (Lennartsson and Rönnstrand, 2012). Signaling *via* c-Kit is required for the maintenance of hematopoietic stem/progenitor cells and their interactions with niche cells (Kimura et al., 2011). Furthermore, gain-of-function and loss-of-function research suggest that even minor changes in c-Kit signaling profoundly affect HSC function (Shin et al., 2014). Dysregulation of c-kit signaling or gain-of-function mutations are strongly associated with tumorigenesis, particularly with gastrointestinal stromal tumors and acute myeloid leukemia (Théou et al., 2004; Wang et al., 2005). On the other hand, c-kit expression is lost in breast cancer (Janostiak et al., 2018) melanoma (Montone et al., 1997), and thyroid carcinoma (Franceschi et al., 2017). Besides the well-established role of mutated/dysregulated c-kit in cancer, current evidence suggests that a functional interaction between c-kit and EPOR increases cancer cell migration, thereby promoting tumor progression (Aguilar et al., 2014). Likewise, its involvement in an autocrine or paracrine growth loop within the tumor microenvironment may represent a molecular mechanism underlying the aggressive metastatic phenotype.

As a classic proto-oncogene mainly mutated or upregulated in cancer cells, c-kit is an attractive target for therapy. So far, great clinical benefits have been achieved from the use of inhibitors of c-kit kinase activity, particularly in patients with gastrointestinal stromal tumors. However, the resistance to c-kit kinase inhibitors occurs with certain oncogenic mutations and can also be developed due to secondary mutations (Ashman and Griffith, 2013). In addition to its well-characterized role as a proto-oncogene, Wang et al. (2018) have recently provided evidence for c-kit tumor suppressor activity. These authors have demonstrated that c-kit can trigger apoptosis in various cancer cells and, more intriguingly, its pro-apoptotic activity could not be detected upon SCF binding, suggesting that c-kit may act both as a proto-oncogene *via* its kinase activity and as a tumor suppressor *via* its dependence receptor activity. Accordingly, the local abundance of SCF is a key factor contributing to c-kit tumor-promoting activity, and therefore CRA-initiated SCF/c-Kit signaling within the tumor microenvironment may stimulate tumor growth and progression.

## Role of Growth Differentiation Factor 15/GFRAL in Impaired Homeostasis and Cancer

GDF15, also named as a macrophage inhibitory cytokine-1, is the TGF-β superfamily protein highly expressed in the placenta and at markedly lower levels in the pancreas, colon, kidney, and prostate. (Rochette et al., 2020). This protein is also selectively expressed in the brain and its expression levels are greatly increased under stress conditions as well as in a number of pathological states including cardiovascular disease, chronic kidney disease, infection, metabolic disorders, neurodegenerative processes, and cancer (Emmerson et al., 2018; Wischhusen et al., 2020). Considering a particular cell type, the expression of GDF15 is frequently detected in adipocytes, macrophages, cardiomyocytes, endothelial cells, and vascular smooth muscle cells, especially upon stressful stimuli (Tsai et al., 2018; Wischhusen et al., 2020). Likewise, GDF15 is expressed in erythroid cells at low levels during normal erythropoiesis, while ineffective erythropoiesis leads to high-expression levels of GDF15 (Tanno et al., 2010). Similar patterns of GDF15 expression under different physiological and respective pathological conditions indicate an adaptive role for GDF15 in response to disturbed tissue/cellular homeostasis.

Binding to a recently identified high-affinity receptor called glial-derived neurotrophic factor receptor alpha-like (GFRAL), GDF15 plays a prominent role in energy homeostasis (Tsai et al., 2018). Yang et al. (2017) have demonstrated that GFRAL associates with the coreceptor RET to elicit intracellular signaling upon GDF15 binding and pointed toward an essential role for this GDF15/GDF15 axis in the reduction of food intake and regulation of body weight in obese mice. Furthermore, GDF15 has been linked to insulin resistance and circulating levels of GDF15 are increased in obese mice and humans as compared to non-obese controls (Kempf et al., 2012). Obesity is associated with low-grade inflammation and increased plasma levels of GDF15 in obese individuals may reflect an adaptive response to a metabolic stress-induced chronic inflammatory state.

Both a chronic inflammatory state and increased GFD15 levels are commonly associated with cancer (Breit et al., 2011). Thus, the levels of GDF15 are greatly elevated in various types of cancers including breast, ovarian, cervical, prostate, colorectal, gastric, and pancreatic. (Modi et al., 2019). Moreover, since its expression levels are gradually increasing during the cancer progression, GDF15 may serve as a prognostic and predictive marker in cancer patients. Additionally, in some tissues, GDF15 seems to be a reliable biomarker in differentiating benign from malignant lesions, for example, chronic pancreatitis vs. pancreatic adenocarcinoma or benign hyperplasia vs. prostate cancer (Wischhusen et al., 2020). Cancer cell stemness, proliferation, migration, and invasion can be directly affected by GDF15. Although the tumor-suppressive effects of GDF15 have occasionally been reported, especially concerning its pro-apoptotic activity in some cancer cells, the vast majority of results support a tumor-promoting role for GDF15 (Emmerson et al., 2018). Similar to other TGFβ superfamily members, this discrepancy may be due to the tumor suppressor activity of GDF15 in the early stages of tumor formation and its stimulative effect on cancer cell migration and invasion during tumor progression (Lebrun, 2012). Pro-tumorogenic effects of GDF15 have been linked with increased activity of different signaling pathways (PI3K/AKT, MAPK/ERK, TGF-β/SMAD), but apart from mediating cancer-induced cachexia (Suriben et al., 2020), a role for GDF15/GFRAL axis in cancer remains to be determined. In addition to direct effects on cancer cells, GDF15 exerts effects on anti-tumor immunity and/or angiogenesis by modulating the tumor microenvironment (Rochette et al., 2020; Wischhusen et al., 2020). Notably, GDF15 may also contribute to common manifestations of cancer, such as anorexia and CRA, leading to a poorer overall prognosis in cancer patients (Suriben et al., 2020; Wischhusen et al., 2020). Moreover, GDF15 may affect hepcidin levels and functional iron status in these patients, and therefore might be involved in the pathogenesis of CRA (Jiang et al., 2014).

## Conclusion

Anemia is generally recognized as an independent predictor of poor prognosis in patients with cancer. Nevertheless, the biological mechanisms underlying this relationship are still not completely understood and need to be further investigated. Thus, CRA may activate SE pathways, causing a marked augmentation of the erythroid progenitor pool in these patients, and therefore requires special attention. In particular, a growing body of evidence suggests a significant role for both stress erythroid progenitors and activated SE pathways in cancer progression. Despite the activation of SE pathways and the consequent expansion of nucleated erythroid cells, if left untreated, CRA still persists due to dysregulated/ineffective erythropoiesis in cancer patients. Inhibiting these pathways may be a promising therapeutic strategy to reduce the tumor-promoting activity of nucleated erythroid cells, along with the management of CRA. Furthermore, some of key players in SE, such as SCF/c-kit and GDF15, have been shown to modulate the erythroid cell response to differentiation stimuli and counteract erythroid cell maturation (Haas et al., 2015; Ranjbaran et al., 2020). Hence, the inhibition of these pathways, apart from reducing the number of nucleated erythroid cells, may also be beneficial for CRA by promoting erythroid cell maturation in cancer patients. However, although the majority of SE-activated signals and their respective downstream pathways have been reported to exhibit tumor-promoting activities, some of them might have an ambivalent or even suppressive effect on tumor growth and/or progression. Most importantly, the nature and magnitude of these effects critically depend on the local microenvironment and the cell/tissue context. Bearing the above in mind, CRA should not be underestimated and its management requires a personalized approach. Namely, considering all contributing causes and complex mechanisms underlying CRA, it becomes clear that no single treatment will be appropriate (both safe and effective) for all patients. Further studies are urgently needed to fully unravel the mechanisms underlying cancer-promoting effects of nucleated erythroid cells and to identify particular SE pathways as potential biomarkers in selecting the most appropriate CRA treatment for each patient.
